# Imagine beyond: recent breakthroughs and next challenges in mammary gland biology and breast cancer research

**DOI:** 10.1007/s10911-023-09544-y

**Published:** 2023-07-14

**Authors:** Renée van Amerongen, Mohamed Bentires-Alj, Antonius L. van Boxtel, Robert B. Clarke, Silvia Fre, Eva Gonzalez Suarez, Richard Iggo, Martin Jechlinger, Jos Jonkers, Marja L. Mikkola, Zuzana Sumbalova Koledova, Therese Sørlie, Maria dM. Vivanco

**Affiliations:** 1 Developmental, Stem Cell and Cancer Biology, Swammerdam Institute for Life Sciences, University of Amsterdam, Science Park 904, 1098 XH Amsterdam, the Netherlands; 2 Laboratory of Tumor Heterogeneity, Metastasis and Resistance, Department of Biomedicine, University of Basel and University Hospital of Basel, Basel, Switzerland; 3 Manchester Breast Centre, Division of Cancer Sciences, School of Medical Sciences, University of Manchester, Manchester, UK; 4 Institut Curie, Genetics and Developmental Biology Department, PSL Research University, CNRS UMR3215, U93475248 InsermParis, France; 5 Transformation and Metastasis Laboratory, Molecular Oncology, Spanish National Cancer Research Centre (CNIO), Madrid, Spain; 6 Oncobell, Bellvitge Biomedical Research Institute (IDIBELL), L’Hospitalet de Llobregat, Barcelona, Spain; 7 INSERM U1312, University of Bordeaux, 33076 Bordeaux, France; 8 Cell Biology and Biophysics Department, EMBL, Heidelberg, Germany; 9 Molit Institute of Personalized Medicine, Heilbronn, Germany; 10 Division of Molecular Pathology, Oncode Institute, Netherlands Cancer Institute, Plesmanlaan 121, 1066CX Amsterdam, The Netherlands; 11 Institute of Biotechnology, HiLIFE Helsinki Institute of Life Science, University of Helsinki, P.O.B. 56, 00014 Helsinki, Finland; 12 Department of Histology and Embryology, Faculty of Medicine, Masaryk University, Kamenice 5, 625 00 Brno, Czech Republic; 13 Department of Cancer Genetics, Institute for Cancer Research, Oslo University Hospital, Oslo, Norway; 14 Cancer Heterogeneity Lab, CIC bioGUNE, Basque Research and Technology Alliance, BRTA, Technological Park Bizkaia, 48160 Derio, Spain

**Keywords:** Lineage tracing, Single cell analyses, Model systems, Breast cancer heterogeneity, Treatment and recurrence, Prevention and early detection

## Abstract

On 8 December 2022 the organizing committee of the European Network for Breast Development and Cancer labs (ENBDC) held its fifth annual Think Tank meeting in Amsterdam, the Netherlands. Here, we embraced the opportunity to look back to identify the most prominent breakthroughs of the past ten years and to reflect on the main challenges that lie ahead for our field in the years to come. The outcomes of these discussions are presented in this position paper, in the hope that it will serve as a summary of the current state of affairs in mammary gland biology and breast cancer research for early career researchers and other newcomers in the field, and as inspiration for scientists and clinicians to move the field forward.

## Introduction

The mammary gland is an intricate organ responsible for lactation and nurturing of newborn mammalian offspring. However, it is also particularly susceptible to carcinogenesis and breast cancer continues to be one of the most prevalent and challenging health concerns worldwide, affecting millions of women. The last decade has yielded many new insights into the complex mechanisms underlying normal mammary gland development and breast tumorigenesis, but an even greater number of questions remain to be resolved.

For example, breast cancer incidence is affected by genetic factors as well as a range of non-genetic factors including steroid hormones, which are essential regulators of normal mammary gland development and physiology [1]. However, the relationship between hormones and breast cancer is complex and not fully understood. Excessive exposure to ovarian hormones has been associated with increased risk of developing breast cancer. In postmenopausal women, circulating estrogen levels have been linked to an increased risk of breast cancer [2] and to a less aggressive tumor phenotype [3]. Other members of the family of nuclear steroid hormone receptors, namely receptors for progesterone, glucocorticoids and androgens have also been implicated in breast cancer development and progression [4–8]. Patients with hormone receptor-positive breast cancer (comprising > 70% of all cases) have a better prognosis than those with hormone receptor-negative tumors. On the other hand, hormone-dependent breast cancer results in a greater risk for long-term relapse that can sometimes occur decades after initial diagnosis [9].

Hence, it is clear that more research is needed to further our understanding of breast tissue in the healthy and diseased setting, specifically concerning *(i)* the molecular mechanisms underlying hormone receptor signaling and (crosstalk with) local, paracrine signaling pathways, *(ii)* the response of the different mammary epithelial and stromal cell types to these long-range and short-range signals at different developmental timepoints and menopausal stages, *(iii)* the impact of (epi) genetic heterogeneity on cell identity, lineage fidelity and plasticity, *(iv)* the response to treatment and the development of resistance, *(v)* the mechanisms underlying breast cancer dormancy, metastasis, and relapse, and finally (*vi*) the impact of lifestyle factors.

## Five breakthroughs of the past decade

The 13 ENBDC committee members quickly reached a consensus on the greatest breakthroughs of the last decade. While some implicit bias cannot be excluded, there was unanimous agreement that the development and application of new technologies, in combination with key biological and clinical research questions, were the driving force behind new biomedical insights in most of these cases. Below, we highlight these breakthroughs, in no particular order.

### Lineage tracing

While the 2000s saw the identification of the mammary stem cells, or more precisely the mammary repopulating units (often abbreviated as MRUs), as a subset of basal cells able to regenerate the entire mammary gland upon transplantation at limiting dilution [10, 11], the next decade challenged and changed our view of what defines a stem cell thanks to in situ lineage tracing experiments carried out in genetically engineered mouse models (GEMMs). The initial promise of these studies was that it would allow us to finally define the cellular hierarchy in the mammary epithelium. While tracing experiments have certainly begun to reveal some of the underlying principles and general rules for how the tissue is organized, especially when combined with mathematical modeling [12], they have first and foremost revealed considerable complexity and plasticity.

Inducible lineage tracing was late to arrive on the mammary gland scene as similar setups had been used successfully in the field of skin development and intestinal homeostasis for quite some time. Here, the fact that tamoxifen is typically used to induce *Cre/lox* mediated recombination of a reporter allele (which forms the basis for most lineage tracing studies to date) deserves special mention. After all, tamoxifen is an estrogen receptor (ER) antagonist that has been shown to affect stem cell content in the human breast [13]. High doses of tamoxifen also delay mammary gland growth during puberty, meaning that care should be taken when designing, executing and interpreting these experiments [14, 15].

The first lineage tracing studies in mice [15–19] were of a qualitative nature, using specific promoters to label defined populations of cells. Some of these were lineage markers (for instance *Krt14* and *Krt8* for basal and luminal cells, respectively). Others were designed to label a specific population of stem cells (e.g. WNT-responsive stem cells marked by *Axin2*^*CreERT2*^) or other cell subsets (e.g. specific luminal progenitors with *Notch2*^*CreERT2*^* or Notch3*^*CreERT2*^). It was not until later that the studies became more quantitative, thanks to the use of multicolor reporter alleles, doxycycline-inducible models and the inclusion of statistical modeling [20, 21].

Collectively, the high number of lineage tracing studies performed to date provide strong evidence that under homeostatic conditions, the basal and luminal epithelial layers of the postnatal mouse mammary gland are maintained by unipotent progenitors, although rare bipotent *Procr* expressing progenitor cells have been reported [22]. Cells expressing PROCR or part of a mouse-derived *Procr* gene signature have also been identified in the human breast [23, 24], but if and how they contribute to tissue homeostasis remains unknown. Unipotency has also been shown for the ERα-positive (ER^+^) and -negative (ER^−^) luminal subsets, which represent two independent and self-sustained lineages [25, 26]. Of note, unbiased or neutral lineage tracing studies that allow either the use of low levels of tamoxifen (in combination with a ‘generic’ driver such as *Rosa26*^*CreERT2*^) or that bypass the use of a chemical inducer altogether, by using sporadic slippage in the *Rosa26*^*[CA]30*^ model, have since confirmed these findings [21, 27, 28].

Several observations are seemingly at odds with this strict separation of basal and luminal progenitors, such as the finding that mammary epithelial cells harbor tremendous plasticity and that bipotency can be reactivated under certain circumstances. For example, transplantation unlocks a regenerative potential in unipotent basal and luminal cells that does not appear to be used under normal physiological conditions, when it is apparently kept in check by cell-to-cell communication between the basal and luminal compartments [17, 26, 29, 30]. Similarly, oncogenic mutations, activation of “stemness” pathways or paracrine signaling from senescent cells can induce features of multipotency in both basal and luminal cells [31–33]. Each of these facultative behaviors may reflect (partial) dedifferentiation to an embryonic state, as the fetal mammary anlage is formed by multipotent precursors. Lineage restriction converts these multipotent cells into unipotent progenitors during later stages of embryonic development [34], but how multipotency is restricted or induced in each of these settings remains poorly understood.

If and how these findings translate to the human breast remains to be determined. Here too, there is some evidence of region-specific, multipotent progenitors [35], but for obvious reasons the options for in situ lineage tracing in humans are limited. Some efforts have been made using sporadic mutations in the mitochondrial genome to identify rare clonal events [36], but these studies are far from painting a comprehensive picture. Plasticity has also been observed in human breast cancer stem cells (CSCs), enabling them to transition between epithelial-like and mesenchymal-like states regulated by tumor cell-intrinsic mechanisms [37], the tumor microenvironment [38] or a hypoxic niche [39]. These transitions may reflect the CSC behavior, as observed by elegant intravital lineage tracing experiments in unperturbed mouse mammary tumors [40].

Taken together, as in most other tissues [41–43], mammary stem and progenitor cell behavior can vary depending on the cell state as well as the spatial, environmental and temporal context. Unfortunately, the in situ visualization of homeostatic cell turnover as well as early, oncogene-induced changes in cell behavior are impeded by the rare nature of these events. Moreover, most clonal tracing analyses have been limited to the static analysis of fixed tissues with a single endpoint analysis per animal and therefore, the dynamic changes in developmental cell state and facultative or oncogene-induced cell plasticity remain poorly understood. Thus, a need remains for real-time, unbiased in vivo lineage tracing experiments to reveal how tracked cells maintain or alter their fate, position and behavior. Ongoing developments in intravital imaging and the use of explant cultures in combination with time-lapse microscopy promise increased temporal resolution. Combining these approaches with unbiased marking of individual cells using unique, heritable barcodes, will allow the simultaneous attribution of clonal fate and gene expression in a time-resolved manner [44].

### Single cell analyses

While tracing experiments essentially collapse a continuum of cell states into a binary event (i.e. a cell either recombines a reporter allele or not), single-cell RNA sequencing (scRNAseq) data have revealed more continuous lineage differentiation trajectories and more complexity than previously appreciated. Of course, this particular breakthrough is not unique or specific for breast (cancer) research. Most biologists have become familiar with t-SNE and UMAP plots for dimensionality reduction, allowing cells to be grouped according to their gene expression profiles. Other single cell omics (ATACseq, ChIPseq, etc.) and multiomics technologies (to obtain simultaneous RNA expression and epigenetic information from the same individual cell) will provide further insight into cell state and cell fate decision making [45, 46].

After publication of the first comprehensive scRNAseq studies of the mouse mammary gland in 2017 [47, 48], the field has expanded rapidly. As a result, we now have access to combined single-cell gene expression and chromatin accessibility profiles of basal and luminal mouse mammary epithelial lineages [49–51], human lactating epithelial cells [52, 53], normal and (pre-)cancerous human breast cells [23, 54], as well as fibroblasts and immune cells in the breast tumor microenvironment [55–57]. Single-cell sequencing also yielded an unprecedented view of the cellular heterogeneity underlying metastasis and resistance to treatment [58, 59]. Interestingly, computational analysis of scRNAseq data has also suggested the presence of bipotent progenitors in the human breast [60], although this still awaits experimental validation.

At present, lineage trajectories inferred from single-cell sequencing data have the downside of losing spatial information as, inevitably, tissue dissociation is necessary to obtain single cell suspensions for these molecular analyses. Ideally, one would be able to overlay the transcriptomic profile with the physical location of a cell. Given that mammary gland branching morphogenesis is non-stereotypic and human ductolobular morphology is diverse, this will likely have to wait until spatial transcriptomics becomes feasible at subcellular resolution.

In mouse mammary tumor models, scRNAseq and scATACseq can be combined with the aforementioned barcode-based lineage tracing to assess clonal dynamics, tumor heterogeneity, clonal competition, as well as to provide insights into the cell of origin and oncogene-induced plasticity. In human cancer, single cell sequencing studies are currently the best way to infer the hierarchical organization and clonal history of tumors, based on the accumulation of mutations in different tumor cell clones [61]. However, these approaches indirectly infer clonality using algorithms affected by clone size, copy number variations, sequencing coverage, biopsy sample and low tumor purity. Thus, at present these methods are useful for comprehending the molecular landscape of breast cancer, but they largely lack lineage information. Moreover, inferring the age of a clone by its mutational repertoire may be complicated by the highly heterogeneous nature and genomic instability of different tumor cells [62–65].

So far, many of these single-cell studies remain largely descriptive and have yielded limited novel insights. While the Human Breast Atlas aims to arrive at an integrated picture [66], there is certainly room for the larger community of breast (cancer) researchers to join forces. Ultimately, multidisciplinary approaches are needed to develop best practices and to interpret the vast amount of multimodal data. Only then will we be able to increase our understanding and functional interpretation of the spatiotemporal dynamics and complexity of the (tumor) tissue with the promise of improving breast cancer treatment.

### New models

The 2010s also saw new experimental models being developed, although these have yet to reach their full potential. Probably the greatest progress was seen in the area of breast cancer patient-derived xenografts (PDX) or orthotopic xenografts (PDOX). These new models of breast cancer are closer to patients’ tumors, remain genetically stable over multiple generations, represent many of the human breast cancer subtypes, can give rise to patient derived xenograft organoids (PDXO) and, in many senses, are thus superior to existing cell line models [67–71].

The original moonshot was that these PDX platforms would allow co-clinical trials in which every breast cancer patient would get their own PDX ‘avatar’. This approach might not be achievable for everyone in the long term, due to low take rates and growth rates of some breast cancer subtypes, but a repertoire of different tumor subtypes capturing sufficient diversity of the tumors encountered in the clinic is within reach. Indeed, the concerted efforts of different academic consortia such as the National Cancer Institute (NCI) PDXNet, EurOPDX and the International Breast Cancer PDX consortium have resulted in a (still growing) collection of over 500 stably transplantable PDX models representing all three clinical subtypes of breast cancer (ER + , HER2 + , and "triple-negative" (TNBC) breast cancer) [67, 68]. Many of these models have also been characterized for genomic, transcriptomic and proteomic features, metastatic behavior, and treatment response to a variety of standard of care and experimental treatments. The establishment of these models in breast cancer, particularly for the more indolent hormone receptor positive breast tumors has not been an easy task. Improved immunodeficient mouse strains and transplantation conditions (i.e. intraductal injection of tumor cells) have resulted in increased take rates of ER + breast cancers [72]. Organoid and PDX models produced by academic groups are available to the breast cancer research community (although national legislation may restrict their export) and can be identified online through the patient-derived cancer model finder [73] and the EurOPDX platform (https://www.europdx.eu). In the UK, the Breast Cancer Now tissue bank provides researchers access to primary cells and tissues [74].

The PDX models have been used to study tumor biology, test novel (combination) treatments, identify and validate response biomarkers, and uncover resistance mechanisms, to the point that they have become essential platforms for advancing precision oncology [75]. However, PDX models lack a functional immune system, and even though tumors can be xenografted into humanized mice, they do not fully recapitulate human immunity – thus limiting their application in immune-oncology research [76].

Complementary to PDX models, GEMMs have remained a valuable tool to study mammary gland development and cancer. However, a clear need was stated to expand the (preclinical) model space, given the difficulties for translating discoveries made in GEMMs (including the lineage tracing studies) to the human setting. This may be, in part, due to differences in mammary biology between mice and humans. Additionally, some of the most widely used GEMMs, such as the *MMTV-Wnt1* or the *MMTV-PyMT* models, develop mammary tumors that have no direct human counterpart. To illustrate: *MMTV-PyMT* mice develop hormone receptor-negative papillary carcinomas that are rare in humans [77, 78]. Yet it has been mouse models on which we largely base our understanding of the stem and progenitor cell hierarchy and the cells of origin for breast cancer – and it is from these models that we infer how the human breast tissue develops and behaves.

We still lack a robust and tractable system in which we can experimentally manipulate and study healthy human breast cells in a 3D tissue context. Mouse organoid cultures have been around for decades and as such pre-date the organoid revolution of the past decade [79, 80]. Typically, these mammary organoid cultures were transient and short lived [81–83], but new protocols allow long-term expansion [84] and advanced control over complex branching morphogenesis and lactogenic differentiation [85–93]. In the wake of the global organoid revolution, healthy human breast organoid cultures (started from reduction mammoplasties) did follow suit [94–96]. However, virtually everyone who has ever tried these cultures, agrees that there is room for significant improvements. For example, the fact that most protocols require the addition of more than 10 different growth factors, makes regular passaging far from simple and also bears the risk of activating signaling pathways that may not be physiological and can bias results. Also, for some of these growth factors, there is little to no understanding of the target cell type and/or the mechanistic effect of the targeted signaling pathway on cell behavior and identity. Moreover, unlike cultures from inbred mouse strains, human breast organoid cultures show considerable variation from donor to donor and even within-donor variation, depending on the vial that is thawed. Efforts to improve these culture protocols are warranted since there is much to gain in the ease with which primary human breast organoids can be used for genetic and pharmacological manipulation. In fact, improvement of organoid technology is likely the only viable option to delineate the dynamics of cell–cell interactions in human breast tissue.

### Tumor heterogeneity

The concept of breast cancer heterogeneity in itself is not new [97], but these days we get to witness this diversity in unprecedented detail. The first reports on the genomic landscape of human breast cancers illustrated the considerable heterogeneity in individual tumors. The identification of the intrinsic molecular breast cancer subtypes (HER2 + , luminal A, luminal B, basal-like and normal-like) based on gene expression patterns [98, 99] and the prognostic gene signatures that separated low from high-risk breast cancers [100–102] initiated an era of high-throughput studies on the molecular profiles of breast cancers. The METABRIC study [103] profiled nearly 2000 human breast cancers and used combined clustering of DNA copy-number variation and transcriptomics to identify 10 new subgroups (or integrated clusters) that only partially overlapped with the previously identified intrinsic subtypes [98, 99]. This rich data source, and others that followed [104–108], are far from being exhausted and can be expected to be a source for hypothesis generation for years to come.

The association of distinct molecular breast cancer subtypes with clinical outcomes [99, 101] provided new opportunities for tumor classification and prognostic tools. The use of such data has now been incorporated into clinical decision-making. In multiple countries, gene expression-based assays such as Prosigna (which uses the 50 genes known as the PAM50 classifier [100, 109], Blueprint (based on an 80-gene signature [110]), Oncotype DX (a qRT-PCR based assay that uses 16 prognostic and 5 reference genes [111]) and Mammaprint (a customized microarray based on a 70-gene signature [101, 112, 113]), are being used to help decide if adjuvant treatments are needed to reduce risk of recurrence after surgery.

Effective treatment is complicated by the fact that tumor heterogeneity does not only occur between patients, but also within a given patient, between primary and metastatic lesions [114], and even within a single lesion [115]. Tumor heterogeneity also evolves during disease progression and treatment [116–118]. As such, heterogeneity affects breast cancer treatment at multiple levels: it complicates diagnosis, makes it hard to identify effective treatment, and it defies therapy, thus leading to resistance [119].

### New drugs have entered the clinic

While the last decade did not see new miracle treatments, multiple new drug development initiatives made their way to clinical care. This is a major improvement after the introduction of endocrine therapy more than 45 years ago, when tamoxifen was approved in the United States for the treatment of metastatic breast cancer. Since then, it has been the first-line endocrine agent for the treatment of ER + breast cancer, contributing to a dramatic reduction in breast cancer mortality [120]. However, a meta-analysis involving almost 63,000 women showed that after 5 years of adjuvant endocrine therapy, breast cancer recurrences continued to appear, with risk increasing over 40% in some cases during a 5–20 year follow-up [9]. This highlights the need for improved therapies that prevent or reduce the development of resistance to current forms of treatment, with the development and use of aromatase inhibitors addressing this concern to some extent [121–123].

While endocrine therapy or anti-HER2 treatment offers specific, guided treatment for a group of patients, patients with TNBC (lacking ER, PR and HER2 expression) still receive (neo) adjuvant chemotherapy. And while this approach is effective in eradicating the primary tumor, it is accompanied by high toxicity, and distant recurrences still arise [124]. Some argued that the introduction of poly(ADP-ribose) polymerase (PARP) inhibitors, which are now also increasingly used for the treatment of BRCA-deficient tumors [125], should be considered a breakthrough. Others held the opinion that PARP inhibitors should still be considered a low-dose chemotherapy, as their working mechanisms are not that different from classical topoisomerase inhibitors [126].

It should be noted that other new treatments, including BCL-2 homology domain 3 (BH3) mimetics, or Phosphoinositide 3-kinase (PI3K) and AKT Serine/Threonine Kinase (AKT) inhibitors, have also been successfully introduced – and these are all examples of targeted therapies that are rationally designed and aimed at specific molecular nodes in the tumor. Cyclin dependent kinase 4 and 6 (CDK4/6) inhibitors, which are now regarded as standard of care for first line advanced ER + /HER2- breast cancer are a good example [127]. At the same time, it should not come as a surprise that, here too, intrinsic and acquired resistance to treatment is being encountered [128].

Stimulation of anti-tumor immunity via immune checkpoint inhibitors (ICI) such as anti-PD-1/PD-L1 and anti-CTLA-4 has proven successful for several tumor types, but results in breast cancer are still modest. Nevertheless, immune checkpoint therapy is bringing hope for some TNBC patients, as shown by the phase III IMpassion130 and phase IIb ALICE trials, but only in combination with a cytotoxic chemotherapy [129–131]. Delineating why some TNBC patients respond and others do not is thus warranted and may lead to improved predictive biomarkers for patient selection. Several combination therapies have been reported to boost the response of TNBC to ICI in preclinical models, but they still require clinical validation [132].

Other developments lie in more refined methods of treating HER2-positive and HER2-low tumors, using bispecific antibodies or antibody–drug conjugates (ADCs) that deliver a chemotherapy payload, such as trastuzumab deruxtecan (T-Dxd) [133–135]. ADCs also hold promise for TNBC [136]. For example, the trophoblast cell surface antigen 2 (TOP2)-directed ADCs Sacituzumab govitecan (SG) and Datopotamab deruxtecan (Dato-DXd) have shown strong and durable responses in patients with metastatic TNBC, resulting in regulatory approval of SG [137]. Other ADCs are likely to follow soon for use in the adjuvant and neo-adjuvant setting in all sub-types of breast cancer.

## Five challenges for the next decade

**Fig. 1:**
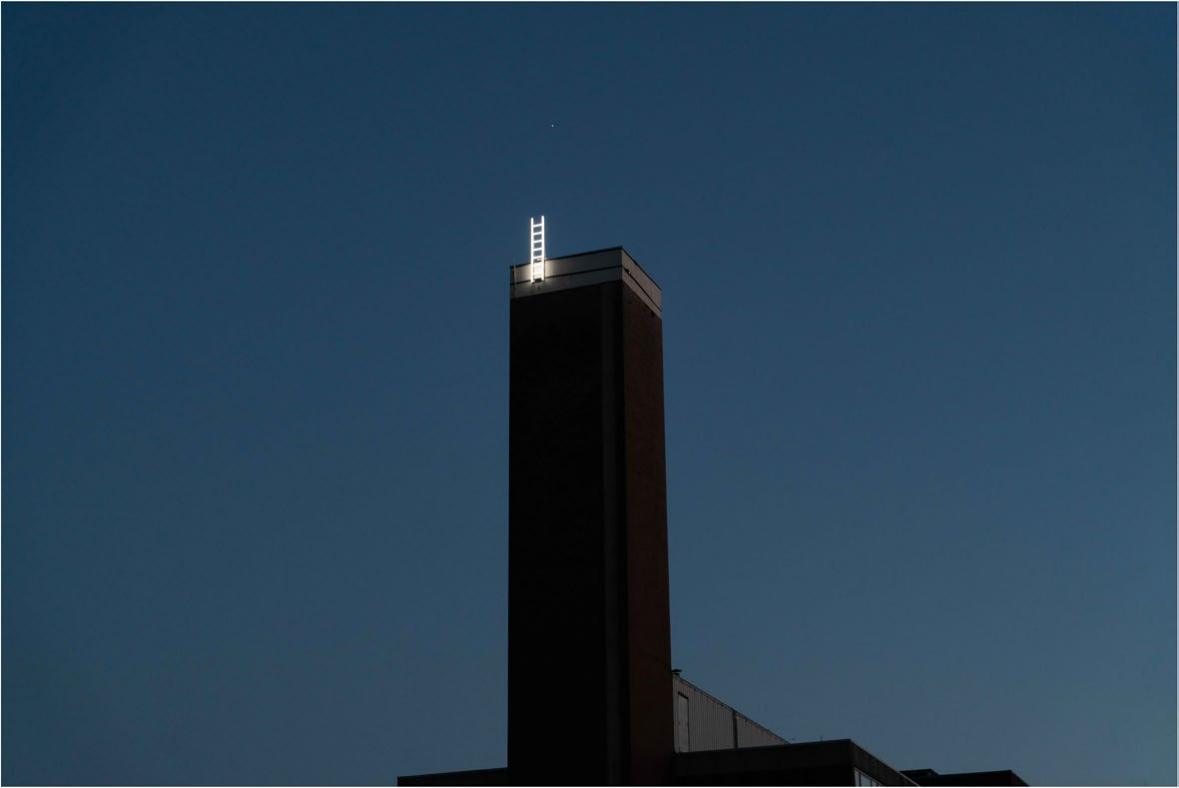
Photo of *Scala a Pioli*, by the Italian artist Massimo Uberti. This piece of light art was on display during the 2022 Amsterdam Light Festival, placed on top of one of the buildings of the University of Amsterdam and seemingly headed into uncharted territory. Image used with permission from the photographer, Sara Kerklaan. This photograph was first published on Folia, the independent online medium for students and staff of the University of Amsterdam

As usual, with every new breakthrough or insight, new challenges arise. Inspired by the theme of the 2022 Amsterdam Light Festival (‘Imagine Beyond’, Fig. 1) we also reflected on the main challenges that lie ahead for our field in the years to come. We identified the following areas in which new discoveries, technological improvements and translational efforts are direly needed.

### Models, assays and platforms

Although this challenge is certainly not unique to mammary gland biology research, the need for faithful models is a key demand for breast cancer. This is due to its notorious complexity resulting from both the inter-tumor and intra-tumor heterogeneity already mentioned above. Patient stratification will become more important as more refined and targeted therapies become available. The lack of realistic models is also a challenge for pre-clinical research and the development of these new treatments. Presently, only about 5% of the anti-cancer agents that undergo preclinical testing are ultimately approved. Many new drugs fail on the way to the market because pre-clinical assays are often a gross misrepresentation of human tumor physiology. Current drug discovery pipelines are still based on simplified tumor cell cultures and animal models, with simplicity, robustness and speed typically trumping relevant complexity and heterogeneity. The time is right to address this gap. So how can and should we account for this in our diagnostic and testing platforms?

We have learnt about the importance of 3D cultures of human breast tissue, both for studying healthy breast and breast cancer biology [94–96]. However, those with hands-on experience agree on the unwieldiness and shortcomings of the current experimental systems. First, the breast (cancer) organoid culture medium is very rich, and yet, the most common ER + tumors often do not grow well under these conditions. Why? Second, possible interactions with inhibitors in the medium need to be carefully considered both when performing drug screens in tumor cultures and when investigating normal breast biology in reduction mammoplasty cultures. The effects of an experimental treatment can be smaller than the inter-donor variation, especially at the transcriptomic level. Other aspects that need to be considered are microenvironment components, including stromal cells, and the extracellular matrix, in addition to the influence of stiffness and other mechanical signals [138]. In the tumor context, alterations in regulatory crosstalk between tumor cells and the tumor microenvironment, with an emphasis on tumor immune surveillance, are especially worthy of attention. In summary, improved human models of normal breast biology and breast cancer are required to develop and select effective treatments for patients, to validate new drugs and to explore the physiology of normal developmental stages in order to improve cancer prevention measures.

At present, the main challenges to improve 3D human breast organoid cultures will be to understand the role of individual growth factors and different extracellular matrix components. In each of these cases, the specific goal should be to maintain cell identity, hormone responsiveness and 3D tissue architecture and function. In addition, can we define more specific culture media for different breast cancer subtypes? Can we generate new cell lines representing all stages and subtypes? As a start, an international effort to generate several normal human breast cell lineages has recently been initiated via the ENBDC. Also, can we develop more long-term faithful (tissue-slice) explant cultures, and use advanced microfluidics for controlled and better engineered microenvironments? Can we use induced pluripotent stem cells as a starting point to understand and then control differentiation and lineage identity of human epithelial and stromal mammary gland cell types? And, when we also consider further improvement of our in vivo models, can we faithfully recapitulate tumor immunity in humanized PDX mice? Development of these new technologies requires significant innovation and multidisciplinary efforts. To arrive at a mechanistic understanding of the processes involved at the molecular and cellular level, the downstream analyses should ideally incorporate artificial intelligence and mathematical modelling to integrate multimodal data and to improve algorithms that extract and combine information from different sources and across different scales to reveal both the governing principles of breast (cancer) biology and grasp the variability that is present in the human population.

### Minimal residual disease and dormancy

Minimal residual disease (MRD) is defined as a reservoir of resistant cells that manage to evade cancer therapy and persist in the tissue for a longer time period before they progress to form a recurrent tumor [139]. Cancer cells can escape dormancy even up to decades after the initial treatment [140]. As such, it is estimated that MRD is the reason behind many incurable relapses, including the aforementioned recurrence of disease after a seemingly complete response to endocrine therapy in ER + patients [9]. The phenomenon of MRD is understudied – and for very obvious reasons: MRD is not accessible for direct functional analysis in patients due to the small number of these cells and the current lack of any clear markers to identify them within the patient tissue.

Due to the cryptic nature of MRD in patients, animal models that faithfully recapitulate human tumorigenesis and permit the study of MRD are the current method of choice to understand mechanisms of recurrence [141]. To follow therapy outcome and tumor regression longitudinally, organoid culture techniques—used in conjunction with GEMMs—have opened up multi-omic analysis avenues for characterizing MRD. The identified molecular hallmarks of MRD could be verified in mouse models and patient samples obtained after neo-adjuvant therapy [142].

From these analyses it has become clear that tumor cell plasticity, rather than specific genetic mutations, define the nature of MRD. This is perhaps not unexpected given the prominent role that non-genetic variation and adaptability play in all aspects of development and disease. It does underscore, however, that to understand tumor cell behavior and oncogenic memory we need to consider various layers of regulation, necessitating the need for more data – from genome-wide DNA methylation analyses to metabolomics.

Of note, while all sparse, remaining dormant tumor cells can be considered MRD, not all MRD cells are necessarily dormant. In fact, some are actively growing and in this case, the immune system seems capable of capturing and killing them [143]. As such, dormancy may be apparent at either the tumor or the cellular level: In tumor mass dormancy, cancer cell proliferation is offset by cell death due to immune surveillance and/or insufficient vascularization without a net increase in tumor cell number [23, 52–54]. True cellular dormancy, on the other hand, occurs when disseminated cells (i.e., cells that have reached a metastatic site) are arrested in the G0 phase of the cell cycle and resistant to host defenses and therapy. Thus, an interplay between reversible quiescence and immune evasion might contribute to MRD surveillance and tumor relapse.

It is important to realize that the same molecular and cellular properties thought to underlie MRD and dormancy can be applied to the concept of cancer stem cells (CSCs). While their role and identity also still remain poorly understood [144, 145], cells with properties of stem cells can resist current forms of therapy, including radiation [146], chemotherapy [147] and hormone therapy [148], facilitating development of tumor recurrence. While it is currently unclear how far MRD and dormancy should be interpreted in this context, as always it is important to let biological understanding and not semantics drive the discussion. In either case, we need a better grasp of oncogenic memory as pertains to the distinct signaling and metabolic states detected in both treatment refractory cells and the initial tumor [149].

What will it take to identify and target these persisting cells? If we cannot eradicate them, can we at least make sure they maintain a state of dormancy? For this, we need to understand what and where these cells are and which mechanisms allow some cells to persist after treatment. Clearly, there are multiple cellular control mechanisms that we do not understand – and therefore our analyses should include all of them; from small RNA species to different posttranslational modifications on histones and signaling proteins. How important is the local niche in the different metastatic sites? Which signals determine if these persisting cells continue to lie dormant or become active again, thus tipping the balance to overt metastasis? What determines the switch between invasive and non-invasive proliferative states? What are the influences of life-style factors in these processes? Answering these and related questions will probably require a combination of organoids from GEMMs and patient organoids that include stromal components (i.e. fibroblasts and immune cells) for mechanistic analyses and serial passaging or transplantation to monitor the tumor initiating capacity typically associated with cancer stem cell identity. In addition, there is a need for longitudinal monitoring of treatment impact in breast cancer patients (for example by measuring cell free tumor DNA), as well as better access to patient material at different stages post therapy including post mortem samples to get insight into MRD – which also requires more sensitive probes and biomarkers to capture tumor cell shedding and monitor MRD.

### Breast cancer as a systemic disease

As is the case with other cancers, breast cancer patients ultimately do not succumb to the primary tumor, but to the burden of distant metastases. For this reason, breast cancer should be seen as a systemic disease. In addition, metastatic niches (in the case of breast cancer these are bone, brain, lung and liver) differ considerably in stiffness and local signaling factors. Here too, the aforementioned intra- and inter-tumor heterogeneity influences seeding and colonization at distant sites. It is a relatively recent insight that the tumor microenvironment, at either the primary or the metastatic site, as well as feed-forward loops between metastatic cancer cells and their microenvironment, also open up opportunities for therapy, including that of tumors that are resistant to immunotherapy [150–152].

One challenging insight is the finding that metastasis can be an early event [153, 154], as recently shown by the identification of mutations in the sodium channel NALCN that promote epithelial cell shedding independently of oncogenic transformation [155]. Early dissemination of disease, as individual (or clusters of) circulating tumor cells, has been detected in the bloodstream and linked to metastatic potential [156]. Of note, increased shedding of tumor cells was recently shown to occur during sleep, with timing of intravasation affecting the metastatic potential of the circulating tumor cells [157]. Systemic signals, including steroid hormone and growth factor signaling, were implied to affect the dynamics of metastasis. This situation could have major implications for clinical treatment regimens, as critical and specific events in tumor biology (such as tumor cell shedding and invasion) should ideally be targeted and treated when they occur.

There are obvious challenges in studying these systemic aspects of breast cancer. First, how do we incorporate them in our experimental models? The circadian rhythm and other aspects of physiology – such as aging, diet, stress and inflammation – can be included as experimental variables in in vivo models – and probably should. At the same time, one might wonder how well these models currently recapitulate human physiology. As just one example, it has been notoriously difficult to model ER + breast cancer in GEMMs, which is why some labs are now – also aided by the developments in genome editing technology – switching to rats [158, 159].

At present, the best we can aim for with our existing human organoid models is to include stromal cell components to model different microenvironments. Other variables are not so easy to implement. For the time being, animal studies are therefore unlikely to be phased out in their entirety. In fact, transgenic mini pigs were also mentioned as a relevant translational model with unmet potential – and many related models could be used and adopted if we broaden our perspective.

In the end, the best model system may be an actual human. Probing individual patients will require more sophisticated liquid biopsy sampling methods and novel biomarkers. With new digital technologies, smart wearables, and a population willing to contribute to citizen science, we are likely to gather more detailed and more diverse metadata related to overall physiology and lifestyle of the general population, as well as the breast cancer-patient population specifically. Ultimately, we might be able to correlate these epidemiological data to breast cancer risk and progression. Proving causality and mechanism, on the other hand, will require an understanding of how these factors interact with a person’s individual (epi)genomic make-up and biochemistry, including hormone biosynthesis pathways. Here, we are challenged with linking these macro-level observations to the as of yet incompletely understood molecular information contained in SNPs and expression quantitative trait loci [160–165]. We are also still in the dark as to if and how the human breast microbiome impacts on the onset and progression of breast cancer [166, 167]. Only once we understand how the interplay between environmental or lifestyle factors and the (epi)genome influence individual breast cancer risk and development, we might truly enter the realm of breast cancer prevention.

### Prevention and early detection

As the saying goes, “prevention is better than cure”. This definitely holds true for cancer, and is the main reason for offering breast cancer screening in high income countries to detect abnormalities as early as possible. The implementation of such nation-wide screening protocols is at least partially responsible for the rise in breast cancer incidence, as it allows early lesions to be detected before they become palpable. While this allows quick and immediate clinical intervention, the question is whether all of these lesions would have progressed to malignant disease. Provided we live sufficiently long, multiple neoplastic lesions will naturally appear in the body as we age [168]. On the other end of the scale, some tumors can escape detection even with these screening protocols in place. Examples are rapidly growing interval breast cancers or tumors that develop in young women who are not yet eligible for screening. So, what determines if and how a tumor develops? Is it the order and the nature of the oncogenic events? The cells of origin? Tumor cell plasticity and/or heterogeneity? The tumor micro- and macroenvironment? Does mild, non-neutral competition play a role in allowing pre-cancerous cells to slowly but surely take over an entire field [169]? If so, what is keeping these cells in check at that stage?

Then there is the very basic question whether all breast cancers arise from luminal cells. No consensus has yet been reached here, although it was widely acknowledged that the luminal progenitor has been crystallizing as the main cell of origin in mouse models of breast cancer. Of note, expressing the PIK3CA oncogene in either basal or luminal cells induced plasticity [32, 33]. The fact that lineage conversion is also known to occur in the presence of epigenetic driver mutations [170], suggests that loss of lineage fidelity may be a prominent step in breast oncogenesis – although it remains to be determined if it necessarily needs to be an early or late event. In either case, it is clear that hybrid cell states exist in breast tumors. We should also consider that even our experimental procedures, such as tissue digestion and cell dissociation, may induce changes in cell state.

Assuming that most human tumors arise from a luminal progenitor, what is the relative contribution of myoepithelial and stromal cells in keeping these cells in check and preventing invasion? In humans, microglandular adenosis has been found to be a non-obligate precursor of TNBC [171]. These rare, preneoplastic lesions have typically lost the myoepithelial cell layer, but still contain a basement membrane. Few ductal carcinoma in situ (DCIS) lesions will transition to invasive breast cancer, and there is still much debate around which criteria should be used to assess the risk for disease progression. At present, some high-risk lesions are still scored as low risk and vice versa, resulting in either under- or overtreatment. Especially for low-risk TNBC, the possibilities for de-escalating chemotherapy should be further studied [172].

Many of these questions warrant a broader societal and political debate, as the classical pathological pipeline is likely to persist. Typically, diagnostics and clinical practice are robust and conservative. Although molecular subtyping assays are increasingly used, and their clinical efficacy is demonstrated through results from multi-year prospective clinical trials such as TAILORx [173], RASTER [174] and MINDACT [175], transcription-based profiling has not become mainstream. The information provided by more traditional means of tumor evaluation (i.e. immunohistochemical and pathological classification) still forms the main basis for selecting therapy in many countries, with the risk of both overtreating and undertreating a subset of breast cancer patients [176, 177]. Challenges therefore remain in not only our understanding of the link (or lack thereof) between defined histological and specific molecular subtypes, but also in making sure that state-of-the-art screening and clinical decision making becomes available across the globe to people of all cultures and backgrounds. In the long term, this will not only improve the quality of life for individual patients, but may also prove to be the most cost-effective [178]. In the end, the added value that will ultimately drive implementation of these new findings is likely to be of economical nature, as observed for the introduction of HPV vaccination in both high- and low-income countries [179–181].

As both the general public and breast cancer patients become more empowered, there will also be a growing role for patient advocates in raising awareness. One example is that a growing number of women know to ask for alternatives to mammographic screening once they become aware that they have dense breasts, in which cancerous lesions are much more difficult to detect. In general, we need to understand more about the biology of breast density, as there is clearly more to the story than difficulties in detection alone [175]. As an obvious aside, there is room for improvement in breast cancer screening methods to begin with: X-ray mammography is effective, but many women will welcome improvements (or alternatives such as tomography or MRI) that make the experience slightly less unpleasant [182].

### Building bridges to cross the divide

While in-depth domain-specific knowledge will continue to be the foundation for future progress, we need to exchange information and collaborate across disciplines in order to flourish. Experimental biologists need to talk to bioinformaticians and computational biologists to make smart use of the vast amount of data that is already publicly available, with a growing need to integrate different data modalities from different platforms. Academic scientists need to meet clinicians, mouse biologists need to talk to those studying the human tissue and basic researchers need to interact with those translating findings to the clinic. This is already becoming increasingly common in many research environments and several European institutes have shared their experiences on how such interactions can be improved [183]. At the same time, these types of multidisciplinary efforts are still not the norm.

For example, the finding that ER + stem cells were found to exist using in vivo lineage tracing experiments in mice, fascinates at least some human biologists since this could provide a possible explanation for the large over-representation of ER + tumors in women. Specifically, long-lived cells are required to accumulate all of the mutations needed to transform a cell. The in vivo lineage tracing studies show that, at least in the mouse, such long-lived, lineage-restricted ER + stem cells really do exist. Virtually all human ER + tumors contain a specific chromosomal rearrangement (der(1;16) or trisomy 1q), caused by recombination between repeats at the centromeres of chromosomes 1 and 16 [184]. If this translocation, possibly induced as a result of hypomethylation of the pericentromeric satellite repeat regions [185], were to bias the lineage to produce more lineage-restricted ER + cells, we would have a plausible explanation for the formation of ER + tumors in humans. Attempts to produce ER + tumors in the mouse by expressing mutant PIK3CA specifically in lineage-traced luminal cells did not produce the human pattern of mainly ER + tumors, but mice do not, and indeed never could, model the der(1;16) translocation because of the different structure of their chromosomes.

We also have lots of work to do when it comes to standardizing (and redefining) our nomenclature. This is especially true now that the developmental complexity of the mouse mammary gland and the human breast are being resolved at single cell resolution [66]. Here, we need to integrate this information with our existing categorizations of different cell populations, which are largely based on FACS sorting and immunohistochemistry. In addition, there is also much to be gained in our understanding of if and how the histopathological definitions and immunohistochemical subtyping (i.e. ‘standard’ staining for ER, PR, HER2 and KI67, sometimes supplemented with KRT5/KRT6 and/or EGFR) relate to the different molecular categorizations. A basic researcher, familiar with molecular subtyping, will likely close *Rosen’s Breast Pathology* [186] feeling overwhelmed and confused by more than 1300 pages of detailed histology. At the same time, they may still occasionally mix up the terms TNBC and basal-like breast cancer, while clinicians are well aware that at least five different subtypes of TNBC exist, each with distinct histological features and opportunities for treatment [187, 188].

Even the simple terms ‘basal’ and ‘luminal’ can be ground for confusion [189]: Histologically, they can refer to the outer (i.e. stroma adjacent) and inner (i.e. lumen adjacent) layers of the mammary gland epithelium, respectively. Often, the word ‘basal’ is used interchangeably with ‘myoepithelial’ – but while all myoepithelial cells are basal, not all basal cells are necessarily myoepithelial. In a human tumor context, however, the term ‘basal-like’ does not reflect the location or for that matter the cell of origin of the tumor (human basal-like breast cancers certainly are not myoepitheliomas). Rather, basal-like breast tumors are classified as such because they (also) express genes that are typically active in basal, rather than luminal mammary epithelial cells (e.g. KRT5 and KRT17) [99]. The terminology of ‘basal’ (i.e. typically expressed by basal cells in stratified epithelia) and ‘luminal’ (i.e. typically associated with simple epithelia) keratins goes back multiple decades [190]. Of note, while many GEMMs use basal (*Krt14* or *Krt5*) and luminal (*Krt8)* promoters to specifically mark the respective outer and inner layers of the mammary epithelium, keratin staining in the human breast is not sufficient to distinguish basal and luminal cells and additional staining with a specific basal marker such as TP63 and a luminal marker such as MUC1 should be included [191, 192].

## Summary and outlook

Ultimately, all of the aspects discussed above need to be combined if we are to build a platform for clinical decision making based on which we can link the tumor genotype and/or phenotype to a specific response to treatment – and offer the appropriate treatment to that specific patient in turn. As an integral and essential part of this, it also remains critical that we continue to invest in studying normal mammary gland biology, both in humans and in other species, as this will be our ‘ground truth’ when it comes to understanding the wiring of gene regulation, cell–cell interactions, basic principles of branching morphogenesis, the cellular mechanisms driving mammary morphogenesis, and the biochemical and metabolic activity of cells along the different lineage differentiation trajectories and in different regions of the tissue. Here, we are about to enter an exciting era as spatial transcriptomics and proteomics are reaching subcellular resolution. In parallel, new high-resolution imaging techniques allow visualization of cellular behaviors at an unprecedented detail under physiological conditions [193]. As such multi-scale, systems-level analyses truly are on the horizon. Finally, where possible, the effects of reproductive history and current reproductive status should be included as they impact on every aspect of breast biology, including the crosstalk between the epithelium and the stroma.

On a day-to-day basis, it can be difficult to see and appreciate the advances being made. Reading the literature on the history of mammary gland biology research ([194, 195] and other reviews or books), or watching interviews with key figures in mammary gland biology and cancer research (https://enbdc.org/interviews/) may help appreciate the tremendous progress made over the years. As the evening fell over Amsterdam, an atmosphere of hope and excitement penetrated our discussions as we pondered the possibilities of using RNAs in a treatment, or anti-inflammatory cytokines in a prevention setting. We also marveled at the recent developments in biotechnology and protein structure predictions, opening up the possibility of developing breast cancer vaccines against neo-antigens. It was also felt, however, that there are distinct difficulties in getting new concepts into treatments or tested in clinical trials. Basic scientists may focus on rapid dissemination and not care about the protection of intellectual property, but it is important if they aspire to bringing their findings to the market. At present, most drug development efforts and clinical trials are run by companies. Clearly, big pharma has its own agenda, which includes the tendency to drop any further investment in drugs once the license expires and with little interest in testing known active drugs in new clinical trial settings (e.g. on different patient groups after improved stratification or in combinatorial treatment regimens based on new molecular insights). This begs the question if we should not uproot the system. Here, individual scientists need a larger infrastructure and support from national funding bodies to reduce barriers. One exciting example is the Cancer Research Horizons initiative, backed by Cancer Research UK, which brings together scientists and other partners in an effort to speed up and increase the translation from bench to bedside. This has already resulted in more than 60 spin out companies and 11 new drugs that have been brought to the market [196].

If these developments show anything, it is that it is key to remain open minded and to continue to promote discovery and push dissemination of novel ideas. Whatever happens in our area of research, much of it will continue to be driven by new technologies and experimental approaches. This piece would therefore not be complete without a plea for sufficient funding for basic scientific research as this is what ultimately drives innovation. Evidently, this should happen in parallel to promoting interactions between basic and translational scientists, clinicians, drug developers and patient advocates. As the ENBDC, we will continue to do our part.
